# Selection of high‐quality and viable blastocysts based on timing of morula compaction and blastocyst formation

**DOI:** 10.1002/rmb2.12302

**Published:** 2019-10-01

**Authors:** Yoshihisa Harada, Tomoyo Maeda, Emi Fukunaga, Reiko Shiba, Shinichiro Okano, Masayuki Kinutani, Toshitaka Horiuchi

**Affiliations:** ^1^ Kinutani Women’s Clinic Hiroshima Japan; ^2^ Graduate School of Comprehensive Scientific Research Prefectural University of Hiroshima Hiroshima Japan

**Keywords:** blastocyst quality, compaction, intracytoplasmic sperm injection, time‐lapse imaging

## Abstract

**Purpose:**

The time‐lapse system is a device that allows continuous monitoring without removing embryos from the incubator. Using a time‐lapse system, we retrospectively investigated cleavage speed time points as potential indicators for selecting high‐quality viable blastocysts.

**Methods:**

This study included 963 zygotes of two pronuclei retrieved from 196 patients between January 2015 and December 2016. All embryos in culture were monitored by time‐lapse after intracytoplasmic sperm injection. Of 492 blastocysts developed in vitro, 128 vitrified‐warmed single blastocyst transfers were classified into pregnancy and non‐pregnancy groups, and the parameters were compared.

**Results:**

In the pregnancy group, timing of both morula compaction and regular blastocyst formation was significantly faster than in the non‐pregnancy group. Furthermore, the optimal cutoff values for compacted morula (94.9 hours) and regular blastocyst (113.9 hours) were determined using the receiver operator characteristic curve analysis. Embryos that formed compacted morulae within 94.9 hours and developed into regular blastocysts within 113.9 hours were associated with a significantly higher pregnancy rate than those that did not (44.4% vs 16.0%).

**Conclusion:**

The timing of morula compaction and regular blastocyst formation is important as an indicator of high‐quality blastocysts to increase odds for pregnancy after embryo transfer.

## 1 INTRODUCTION

In many in vitro fertilization (IVF) clinics, blastocyst transfers have been performed that involve selection of high‐quality embryos, leading to improved implantation rates. In the past, with assisted reproductive technology (ART), pregnancies with multiples have been a serious problem resulting from transfer of several embryos to the uterus,^1^, ^2^ but single blastocyst transfer reduces this risk dramatically. Obtaining blastocysts for embryo transfer requires that embryos be cultured for 5‐6 days in vitro.^3^ If multiple blastocysts are obtained from in vitro culture, high‐quality blastocysts must be selected for the best odds of continued embryo fetal development.

Widely used indicators for evaluating blastocyst quality based on developmental stage are the scale of the inner cell mass (ICM) and trophectoderm (TE) cell number.^4^ These are morphological evaluations, however, and carry the risk of subjectivity. Objective indicators are needed for better predictive criteria for selecting high‐quality blastocysts in ART. In recent years, the usefulness of a time‐lapse observation system during embryonic development has been reported.^5^, ^6^, ^7^ The time‐lapse system allows for monitoring of embryo development at all times without removal of the culture dish from the incubator. Using a time‐lapse system, the morphokinetics of embryos and the division speed both can be measured. This approach thus enables the noninvasive objective assessment of high potential embryos and provides a tool for predicting embryo development and implantation.

Many reports have demonstrated the usefulness of time‐lapse, but several of these studies have predicted embryo development in early cleavage,^8^, ^9^, ^10^, ^11^ with few reports addressing blastocyst selection from in vitro culture.^12^ In the embryonic developmental process, compaction of the embryo has an important influence on blastocyst and ICM formation and TE differentiation. The relationship between the precise timing of compaction and blastocyst formation and their mechanisms is not clear. In this study, by targeting embryos fertilized by intracytoplasmic sperm injection (ICSI) for which the sperm injection time is clear, we predicted that the time from cleavage to embryo development could be measured accurately. The purpose of this study was to establish an objective indicator of high‐quality blastocysts using time‐lapse monitoring. We retrospectively evaluated the relationship between compaction and blastocyst formation timing based on comparison of data from pregnancy and non‐pregnancy groups.

## 2 MATERIALS AND METHODS

### 2.1 Patients

This study used two pronuclei from each of 963 zygotes fertilized by ICSI, obtained from 194 patients from January 2015 to December 2016. The age (mean ± standard deviation) of patients at the oocyte retrieval cycle was 38.3 ± 4.3 years (range: 26‐49). All embryos were monitored by a time‐lapse system (Primo Vision; Vitrolife). In addition, we examined the outcome of 128 cycles of vitrified‐warmed single blastocyst transfer obtained from the same cycles. This retrospective study was approved by the Ethics Committee of Kinutani Women's Clinic, Hiroshima, Japan.

### 2.2 Stimulation protocols and oocyte retrieval

Women were treated with GnRH agonist using either a short protocol or long protocol and with GnRH antagonist, according to each patient's ovarian response and medical history of IVF treatment. In some cycles, the minimal stimulation protocol was used, with a combination of low‐dose gonadotropin injection and clomiphene citrate. When a follicle reached a mean diameter of ≥18 mm, human chorionic gonadotropin (Aska Pharmaceutical) at a dose of 5000 or 10 000 IU was administered. Cumulus‐oocyte complexes were collected 35 hours after human chorionic gonadotropin administration using transvaginal ultrasound‐guided puncture of the follicles. Collected cumulus‐oocyte complexes were denuded by pipetting with 80 IU/mL hyaluronidase (Irvine Scientific). The denuded oocyte maturity was assessed by visualization of the first polar body under a stereoscopic microscope. Only MII oocytes were used for ICSI.

### 2.3 In vitro culture of human embryos and analysis of time‐lapse imaging

**Figure 1:**
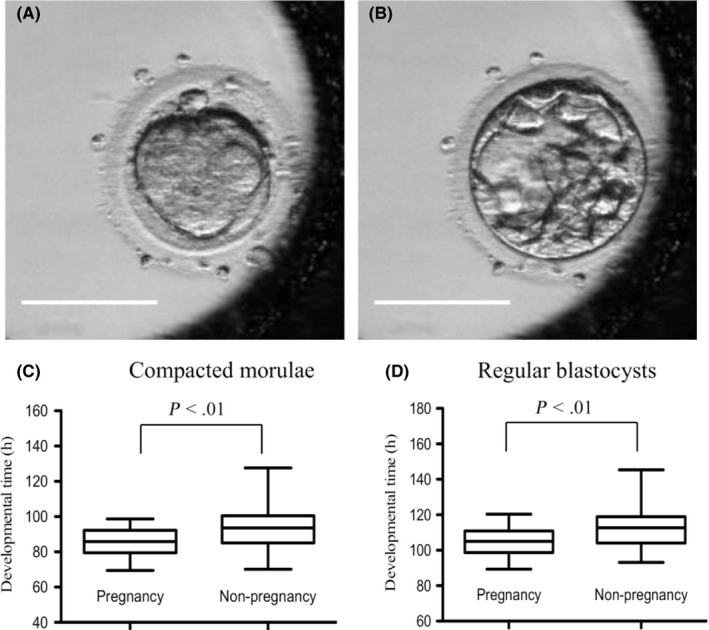
Time‐lapse images of a compacted morula (A) and blastocyst formation (B). (C and D) Developmental timing of the compacted morulae and regular blastocysts, respectively, compared between pregnancy and non‐pregnancy groups after single blastocyst transfer. The timing of morula compaction and regular blastocyst formation was significantly different between the two groups (*P* < .01, Mann‐Whitney *U* test). Bars = 100 µm

Embryos fertilized by ICSI were cultured in WOW dishes (Vitrolife) with SAGE 1 Step (Origio) or continuous single culture medium (Irvine Scientific) at 37.0°C under 6% CO_2_, 5% O_2_, and 89% N_2_ for 5‐7 days and evaluated for development to the blastocyst stage. The oocytes were individually cultured in a humid incubator–equipped time‐lapse system (PrimoVision; Vitrolife). A total of 1‐16 embryos were placed in a 150 μL drop of medium, and images of the embryos were recorded automatically at 10‐minute intervals. The embryos were assessed and analyzed at five time points from the first image recorded of the following events: pronuclei appearance, appearance of male‐female pronuclei in the cytoplasm; and pronuclei breakdown: disappearance of male‐female pronuclei in the cytoplasm completely. Timing of the two‐cell stage was considered as the complete division from one to two cells, and abnormal cleavage (1 to >3 cells) or indivisible blastomeres were excluded from measurement. The time point of morula compaction was considered to be when blastomeres tightened at the morula stage, and individual cell boundaries were considered to be unclear (Figure 1A). The time point of regular blastocyst formation was defined as the presence of a formed blastocoel that increased in volume and filled the embryo completely (Figure 1B).

### 2.4 Blastocyst vitrification, warming, and single embryo transfers

Blastocyst vitrification was done using the method by Hiraoka et al^13^ The blastocysts were placed in equilibration solution containing 7.5% (v/v) EG (ethylene glycol, Sigma‐Aldrich) and 7.5% (v/v) DMSO (dimethyl sulfoxide, Nacalai Tesque Inc, ) in mHTF (Irvine Scientific) supplemented with 20% (v/v) SSS (Irvine Scientific) at 37°C. They then were transferred into vitrification solution containing 15% (v/v) EG, 15% (v/v) DMSO, and 0.5 mol/L sucrose (Sigma‐Aldrich) in mHTF supplemented with 20% (v/v) SSS for 1 minute at 37°C. The blastocysts were loaded onto Cryotops (Kitazato Supply) at a minimum volume and immediately dipped into liquid nitrogen at −196°C. Artificial shrinkage (AS) of blastocoel was performed in the equilibration solution by glass pipetting. After blastocoel collapsed completely, the blastocysts were equilibrated in the equilibration solution another 2 minutes. If blastocoel was not collapsed completely, the blastocysts were equilibrated in the equilibration solution for up to 10 minutes. For warming, the tip of the Cryotop was immersed directly into 1.0 mol/L sucrose solution for 1 minute at 37°C. The blastocyst was transferred to 0.5 mol/L sucrose solution for 3 minutes and washed twice in mHTF supplemented with 20% SSS for 5 minutes at 37°C. The warmed blastocyst was cultured for approximately 1‐4 hours before recovery for embryo transfer with Embryo Glue (Vitrolife) in a humid incubator at 37°C under 6% CO_2_, 5% O_2_, and 89% N_2_ in air. The vitrified‐warmed embryo transfer was performed by using the hormone replacement therapy cycle or natural cycle. A clinical pregnancy was defined as a pregnancy with a gestational sac confirmed in the uterus by ultrasound at 4 weeks after embryo transfer.

### 2.5 Statistical analysis

Pregnancy, abortion, and live birth rates were compared using the chi‐square test. Because the time‐lapse data were not normally distributed, we used the nonparametric Mann‐Whitney U test instead. For comparison between the two groups along normal distribution, we used Student's *t* tests. The statistical analysis was performed using GraphPad PRISM 6.03 software (GraphPad Inc). The receiver operating characteristic (ROC) curve was created to determine the cutoff value using JMP 14.0 (SAS Institute, Inc). Significant differences were assumed to be present at *P* < .05.

## 3 RESULTS

### 3.1 Comparison of developmental time between the blastocyst and arrested embryo groups

In this study, 492 out of 963 (51.1%) embryos from ICSI reached the blastocyst stage (blastocysts group), and 471 (48.9%) embryos were arrested in vitro (arrested embryos group). The results of cleavage speed are shown in Table 1. The timing of pronuclei appearance was not significantly different between the two groups (9.1 vs 9.3 hours). However, the timing of pronuclei breakdown in the blastocyst group was significantly faster than in the arrested embryos group (22.2 vs 24.0 hours; *P* < .01). Moreover, the time to the two‐cell stage in the blastocyst group was significantly faster than in the arrested embryos group (25.0 vs 26.5 hours; *P* < .01), as was the time to the compacted morula (90.4 vs 96.8 hours; *P* < .01). In the blastocyst group, the timing of regular blastocysts was 112.5 hours after ICSI.

**Table 1:** Comparison of developmental timing between human blastocysts and arrested embryos groups by analysis of time‐lapse monitoring

Total no. of pronuclei embryos	963		*P*
Developmental groups	Blastocysts	Arrested embryos	–
No. of embryos (%)	492 (51.1)	471 (48.9)	
Developmental time (h)			
Pronuclei appearance [range]	9.1 [4.0‐16.7]	9.3 [4.4‐17.9]	NS
Pronuclei breakdown [range]	22.2 [16.5‐34.8]	24.0 [16.3‐56.7]	<.01
Two cells [range]	25.0 [18.7‐38.2]	26.5 [19.2‐51.8]	<.01
Compacted morula [range]	90.4 [54.9‐129.7]	96.8 [68.5‐142.1]	<.01
Regular blastocyst [range]	112.5 [85.4‐166.8]	–	–

Abbreviation: NS, not significant.

### 3.2 Comparison of developmental time between pregnant and non‐pregnant groups after single blastocyst transfer

The cleavage speed of the transferred blastocyst was compared with or without pregnancy (Table 2, Figure 1C,1). Between the two groups, time to the two‐cell stage did not differ (23.2 vs 23.8 hours). However, the times to a compacted morula and regular blastocyst (86.1 vs 93.2 hours; *P* < .01 and 105.0 vs 113.0 hours; *P* < .01, respectively) leading to pregnancy after single blastocyst transfer were significantly faster than those of embryos that did not result in pregnancy.

**Table 2:** Comparison of developmental timing between pregnancy and non‐pregnancy groups after single blastocyst transfer, by analysis of time‐lapse monitoring

Total no. of blastocysts transferred	128		*P*
Pregnancy	+	−	–
No. (%) of blastocysts	41 (32.0)	87 (68.0)	
Developmental time (h)			
Two cells [Range]	23.7 [19.4‐30.8]	24.6 [18.7‐31.6]	NS
Compacted morula [range]	86.1 [69.4‐98.7]	93.2 [70.2‐127.7]	<.01
Regular blastocyst [range]	105.0 [89.3‐120.3]	113.0 [93.2‐145.4]	<.01

Abbreviation: NS, not significant.

### 3.3 Clinical pregnancies and live births based on time‐lapse selection criteria: morula compaction within 94.9 hours and regular blastocyst formation within 113.9 hours

**Figure 2:**
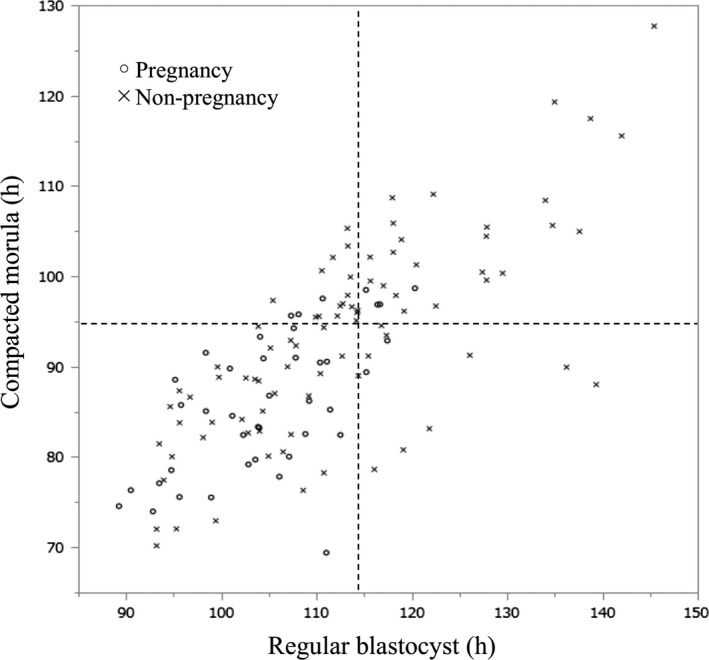
Distribution of pregnancy or non‐pregnancy based on cutoff values of time from intracytoplasmic sperm injection (ICSI) to compacted morula and time from ICSI to regular blastocyst. The AUC is 0.71. AUC, area under the receiver operator characteristic curve

Distribution of pregnancy or non‐pregnancy was based on cutoff values of time from ICSI to compacted morula and time from ICSI to regular blastocyst (Figure 2). The optimal cutoff values for compacted morula (94.9 hours) and regular blastocyst (113.9 hours) were determined from the receiver operator characteristic curve. Table 3 shows the pregnancy, abortion, and live birth rates after single blastocyst transfer. When single transfer blastocysts developed to the compacted morula stage within 94.9 hours and reached the regular blastocyst stage within 113.9 hours after culture, the clinical pregnancy rate (44.4%) was significantly higher than for blastocysts that did not meet these criteria (16.0%). Similarly, the live birth rate in the former group was significantly higher than in the group associated with blastocysts that did not meet these criteria (37.5% vs 10.7%; *P* < .01).

**Table 3:** Clinical pregnancies and live births based on time‐lapse selection criteria: morula compacted within 94.9 h and regular blastocyst formation within 113.9 h

Time‐lapse selection criteria	Applicable	Not applicable	*P*
No. of single blastocysts transferred	72	56	–
No. of clinical pregnancies (%)	32 (44.4)	9 (16.0)	<.01
No. of abortions (%)	5 (15.6)	3 (33.3)	NS
No. of live births (%)	27 (37.5)	6 (10.7)	<.01

Abbreviation: NS: not significant.

## 4 DISCUSSION

The present study showed that morula compaction and blastocyst formation times are important as indicators of high‐quality viable blastocysts that successfully implant and progress developmentally. Especially, we showed that the time of compacted morulae is of importance as the criteria of pregnancy and live birth after embryo transfer. On the other hand, the two‐cell stage time emerged as an index for predicting which embryos will develop into blastocysts. Several studies have reported that early cleaving embryos at the two‐cell stage have higher rates of progressive embryonic development and clinical pregnancy than those involving late‐cleaving embryos.^14^, ^15^, ^16^, ^17^, ^18^, ^19^, ^20^, ^21^ In this study, the time to the two‐cell stage in the blastocyst group was earlier than in the group with arrested embryo development. However, the time to the two‐cell stage in the successful pregnancy group was similar to that in the group that experienced failed pregnancy. The mechanism regulating the timing of the two‐cell stage in humans remains incompletely understood. The state of gene expression suppression before fertilization is uninhibited after fertilization, and the expression of zygotic gene activation from the one‐cell to two‐cell stages may also affect two‐cell stage timing.^22^ In mice, chromosomal abnormalities have been reported to delay DNA replication and alter cell cycle and division length.^23^ Regardless of sperm count, the timing of the two‐cell stage may be related to sperm DNA damage. In the mouse experiment, we have shown that the sperm DNA damage delays the time of two‐cell and the subsequent cleavage time (unpublished data).

As a result of the analysis, using ICSI as the starting point, the time to morula compaction as well as time to blastocyst formation was significantly earlier in pregnancy vs non‐pregnancy groups. These results indicate that the earlier compaction completion and blastocyst formation times are important indicators of embryo quality. That conclusion is consistent with the results of previous studies showing that compaction patterns affect the rate of good‐quality blastocyst retrieval and pregnancy.^24^, ^25^ Campbell et al have been reported that the time to compaction was significantly earlier in euploid vs aneuploid embryos (79.7 vs 85.1 hours).^26^ Assuming that the blastocyst leading to pregnancy was euploid, their results agree in our study. The mechanism linking a high‐quality embryo and early compaction completion is not clear. Transcription of the embryonic genome begins on day 3 and is activated on day 4 physiologically.^27^, ^28^ The intercellular adhesion protein E‐cadherin is expressed during compaction, allowing cells to adhere more tightly.^29^ Several studies have reported the relationship between compaction and embryo quality using time‐lapse monitoring. The pattern of compaction completion time affects the embryo implantation rate^30^ and starting compaction before the eight‐cell stage is usually associated with aberrant embryonic development.^31^

In addition, embryos that progressed in development reached the blastocyst stage early, indicating that embryos that complete compaction early reach blastocyst early, as well. Several reports have addressed the timing of blastocyst formation. In the vitrified‐warmed cycles, results suggest that day 5 and day 6 blastocysts have similar implantation rates.^13^, ^32^, ^33^, ^34^ On the other hand, delayed blastulation is associated with lower live birth rates in frozen cycles.^12^, ^35^, ^36^, ^37^

The cause of delayed blastocyst formation could be the embryo itself or the result of a less‐than‐optimal in vitro environment that negatively influences embryonic development. Indeed, the risk of long‐term in vitro culture of embryos until expansion into blastocysts is well known.

In addition, the case subject to analysis is a vitrified‐warmed cycle. It has been reported that blastocyst survival rate is improved by AS.^13^, ^38^ AS was performed in the equilibration solution by glass pipetting.^13^ By this method, a survival rate of 99% or more and live births has been acquired for several decades, it is possible to think that there is almost no risk of vitrified and warming.

From these results, we can conclude that the timing of morula compaction can be predictive of an embryo's potential to progress to a high‐quality viable blastocyst. However, this study is a retrospective study, and a prospective study is necessary to further clarify the prediction criteria. Time‐lapse monitoring enables continuous observation of embryonic development and is useful for observing embryonic morphokinetics and division speed. Time‐lapse has made it possible to obtain more information on embryo development at different times compared to previous single morphological observations, without removing the embryo from the incubator. In modern ART, objective evaluation indicators such as division speed and morphokinetic parameters may facilitate future IVF success.

In conclusion, the timing of the two‐cell stage emerged as an indicator of the development of high‐quality blastocysts. The timing of morula compaction completion and regular blastocyst development also were important for selection of high‐quality viable blastocysts that progress in development and lead to live birth.

## DISCLOSURES

*Conflict of interest*: The authors declare no conflict of interest. Human rights statements and informed consent: All procedures were followed in accordance with ethical standards of the institutional ethical committee and with the Helsinki Declaration of 1964 and its later amendments. Informed consent was obtained from all patients in this study, and the study design was approved by the ethics committee of Kinutani Women's Clinic, Hiroshima, Japan. Animal studies: This article does not contain any study with animal participants that was performed by any the authors.
